# New perspective in heart failure management: could myosin activators be the answer?

**DOI:** 10.15190/d.2014.25

**Published:** 2014-12-31

**Authors:** Stefan Andrei, Corina Iorgoveanu

**Affiliations:** CNHO des Quinze-Vingts, Service de Médecine Interne, Paris, France; CC Iliescu Institute of Cardiovascular Diseases, Cardiology, Bucharest, Romania

**Keywords:** Heart failure, omecamtiv mecarbil, myosin activator

## Abstract

Heart failure is a worldwide leading cause of hospital admissions. There is a critical need for new methods of management in acute heart failure. The current drug panel available in the treatment of hemodinamically unstable patients is not only limited, but it is also associated with deleterious side effects. Discoveries in the heart failure field seemed to lack an adequate answer and a change in paradigm may be necessary. The cardiac myosin activator omecamtiv mecarbil is a new therapeutic approach that improves the myocardial contractility through an innovative mechanism, avoiding the harmful effects of currently used inotropic agents. Several studies provided us with promising results, but further scientific proofs are needed.

## 1. Introduction

Considerable progress has been made during the last decades in understanding, prevention and management of cardiovascular disorders (CVD), but cardiovascular-related health issues still remain the leading cause of death worldwide, accounting for almost 30% of global deaths^1-3^.

A variety of cardiac diseases can converge in time to heart failure (HF), a pathological condition characterized by heart’s inability to provide oxygen in response to tissular metabolic demands, with normal or increased filling pressures^4^. In the developed world it is estimated that 1-2% of general population have HF^5^. The pathophysiological mechanisms of this condition are complex and involve various adaptive interconnected neurohormonal pathways which enter a positive feedback loop with deleterious long term effects. HF may have a preserved (HFPEF) or a low ejection fraction (HFLEF), according to the pathophysiological stage. The actual therapeutical approach in chronic HFLEF is focused on neurohormonal antagonism with angiotensin enzyme inhibitors, beta-blockers and mineralocorticoid receptor antagonists, associated with preventive measures, risk factors and comorbidities management. Non surgical and surgical supportive devices play also a role^4^.

## 2. Inotropes - currently essential in acute care but avoided on long-term

A pharmacological class of drugs that is traditionally used to improve systolic function in patients with HF and to break the neurohormonal loop are the inotropes (**Table 1**). These agents stimulate heart contractility through various mechanisms, being particularly administered in order to control hemodynamic status in acute settings and to ameliorate symptoms in chronic patients^4,6^.

Cardiac muscle cells have a special design of sarcomeres, where fibers are coupled through intercalated discs which contain gap junctions that are involved in transmitting a depolarizing current along the fibers’ length^7^. According to the model suggested by Andrew Huxley et al., muscle contraction is achieved by myosin and actin sliding movement, a process made possible by ATP hydrolysis. A distinguishing trait of cardiac muscle is that calcium is released from both the sarcoplasmic reticulum and the extracellular matrix by a particular mechanism called calcium-induced calcium release. The action potential generates an influx of calcium through T tubules, which activates ryanodine receptors found on sarcoplasmic reticulum. The cytosolic calcium binds cTnC and subsequently tropomyosin is being shifted, releasing the myosin binding sites on actin^8-11^.When there is no ATP attached, myosin binds firmly to actin. ATP bound to the head of myosin leads to dissociation of myosin-actin complex by inducing a conformational change in myosin. After the ATP hydrolysis, its products -ADP and Pi- continue to be attached to the myosin, forming a very stable complex until the slow dissociation occurs. This last step is rate-limiting in the cross-bridge cycle^8^. During the contraction cycle only few myosins succeed at being recruited^12^.

Inotropic agents work either by augmenting calcium sensitization or by modulating the level of intracellular calcium, a process made possible through two main mechanisms: inhibition of the Na/Ca exchange pump or increasing the level of cAMP. The latter is achieved either by stimulation of adenylate cyclase or by inhibition of cAMP degradation via phosphodiesterase (PDE)^13^*.*

**Table 1:** Current inotropes and their mechanism of action^13^

Agents that increase intracellular cAMP	Agents that affect sarcolemmal ions, pumps and channels	Agents that modulate the release or sensitization of proteins to intracellular calcium	Agents with multiple mechanisms of action
**Catecholamines (Beta Adrenergic Agonists**)**Natural**: *Adrenaline*; *Noradrenaline*;* Dopamine*; **Synthetic**: *Dobutamine*;* Isoprenaline*; *Dopexamine*; Ibopamine*; **Phosphodiesterase inhibitors**: Milrinone; Amrinone; Enoximone*	*Digoxin*	**Increased intracellular Ca release** 1.Flosequinan * **Increased Ca sensitization ** 2.Levosimendan*	**PDE III inhibitor/Ca sensitizer** 1.Pimobendan * **PDE III inhibitor/ modified rectifying** **K currents** 2.Vesnarinone*
* not FDA approved			

The downside of using inotropic agents in the treatment of acute heart failure is their possible adverse events. The main inotropic action mechanism is also the reason of potential development of cardiac arrhythmias^14^. Besides the fact that increased intracytosolic calcium is the main cause to this susceptibility to developing tachyarrhythmias, it also leads to more energy involved into calcium transport back in the sarcoplasmic reticulum through Ca-ATPase (SERCA) pumps, the aftereffect being increased oxygen demand^14-16^.

Another problem that should be taking into account is the possible hypotension, which is the result of increased cAMP in the peripheral vessels, leading to poor coronary perfusion^15-17^. Beta adrenergic agonists work by activating G protein, which has as an end-point increased levels of cAMP, through the action of adenylyl cyclase on ATP. In myocytes, this process leads to phosphorylation of L-type calcium channel and the subsequent influx of calcium activates ryanodine receptors, ultimately increasing Ca-mediated myocardial contractility. In the peripheral vasculature, activation of beta-2 adrenergic receptors leads to vasodilation, a process explained by the phosphorylation of the protein phospholamban, which makes possible the reuptake of calcium by the sarcoplasmic reticulum^6,18^.

Patients with decompensated heart failure, under chronic treatment with full-dose beta blockers, might not experience an adequate increase in cardiac output when dobutamine is administered. Instead, they could be treated with phosphodiesterase inhibitors (PDEI)^19^. On the other hand, PDEI should not be used in patients with reduced glomerular filtration rate, as milrinone is renally excreted^6^. It is demonstrated in OPTIME-CHF (Outcomes of Prospective Trial of Intravenous Milrinone for Exacerbations of Chronic Heart Failure ) study that the use of milrinone is not recommended in patients with decompensated heart failure not only because of tachyarrhythmias and hypotension events, but also for the fact that there is no decrease in the rate of mortality between Milrinone and placebo groups^20^.

Another category of inotropes, calcium sensitizer agents (prototype: Levosimendan), work by improving the binding of Ca to troponin C, independent of adrenergic pathway. For this reason, it seems to have less pro-arrhythmic side effects and does not cause increased oxygen consumption. Moreover, levosimendan appears to cause vasodilation through ATP-sensitive potassium channels, but only at high concentrations, that are not usually used in clinical practice. However, no difference in mortality has been noted between levosimendan and dobutamine. Furthermore, because of its vasodilation effect, calcium sensitizers have not been effectively studied in patients with cardiogenic shock, more clinical trials being required^21^.

## 3. Paucity of new molecules in heart failure treatment

Despite this epidemiological context, there is an important gap in developing efficient new molecular entities (NME) for HF treatment, with no Food and Drug Administration (FDA) approved inotropic agent in the last two decades^22^. Tolvaptan - a selective V2 vasopressin antagonist - is the only HF-related NME approved in the last 10 years but for treatment of hyponatremia, including hyponatremic HF^23^. According to Pierre Pollesello, some contributing factors to this situation might be the continuously evolving HF definition, the low cost for present medical therapies, the redirection of funds towards other research areas by pharmaceutical companies, the demands for expensive long term mortality trials and study recruiting issues related to the heterogeneity of HF patients^24^. This might also imply that we are not looking in the right direction yet and new research perspectives are needed.

## 4. Myosin activators are a possible answer

The discovery of a new class of inotropic drugs appeared to be a possibly feasible step form theory to practice^25^. A small molecule that enhances contractile motor protein myosin activity bypassing the second messenger regulation could bring new hope in HF treatment^26^. Theoretically, an ideal small molecule myosin activator should ameliorate the rate-limiting step in actin-myosin cycle, would recruit more myosin heads during the cardiac circle, would not have vascular effects and would not elicit calcium-related adverse reactions^27^. So, the overall effect would be to improve cardiac contractility in a “more hands on the rope” manner^28^. Several compounds have been developed and tested in preclinical studies deepening our understanding about this inotropic mechanism and about the necessary properties^29,30^.

## 5. The new hope: omecamtiv mecarbil?

**Figure 1:**
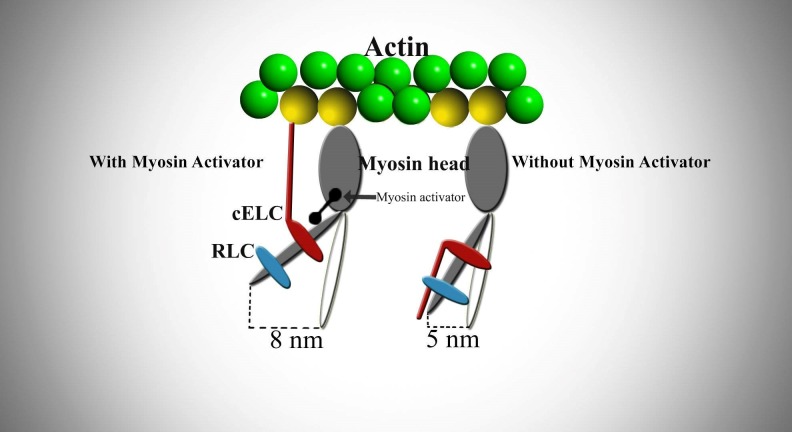
OM does not increase the force of myocardial contraction by altering the calcium concentration, but by acting directly at the level of sarcomere The fast change from weakly bound to the strongly bound actin-myosin configuration activates the sarcomere, increasing the force of cardiomyocyte displacement up to 8nm, compared to 5nm without myosin activator. Two stabilizing light chains - essential (ELC) and regulatory (RLC) - not only hold the neck of the myosin head in a steady position, but are also the mechanical components that are part of the moving force of myosin. These myosin light chains have the particularity of a regularly turning around in order to impel the attached actin, being important controllers of cardiac force production^33^.

A group from Cytokinetics finally succeeded in creating and refining a compound with the needed characteristics for entering the clinical trials step: CK-1827452, AMG 423 or omecamtiv mecarbil (OM)^31^. This new molecule is able to specifically bind at S148 heavy chain activating the cardiac myosin, but it has no effect on the smooth nor the skeletal muscle myosin^32^. OM appears to speed up the transition of myosin from a weak actin-binding in a strong actin-binding force-generating state, this being the rate-limiting step of actin-myosin-ATP interaction and also of myocyte contraction (**Figure 1**)^32^. In vitro, OM increased the duty cycle of acto-myosin interaction in tissue purified ventricular myosin^33^.

The results obtained by using two canine models of HF were promising. The HF was induced by rapid pacing in two situations: after myocardial infarction induced by ligation of left anterior descending coronary artery and after myocardial hypertrophy caused by 1 year - long ascending aortic banding. OM was administered in 0.25 mg/kg bolus followed by continuous perfusion of 0.25 mg/kg/h during 24h. The treatment decreased the heart rate (HR), mean left atrial (LA) pressure, left ventricular (LV) end-diastolic pressure and improved LV systolic ejection time (LVSET), systolic wall thickening and stroke volume (SV) at 15 minutes, 4 hours and 24 hours of treatment and increased cardiac output at 24 hours. A reduction in vascular peripheral resistance was noted at 4 and 24 hours. No changes of LV dP/dt max, mean arterial pressure or myocardial oxygen consumption were remarked^34^. Multiple studies in humans were performed, they are listed in **Table 2**.

**Table 2:** Omecamtiv mecarbil – human clinical studies

Clinicaltrials.gov identifier	Study	Design	Status	Data	Results
NCT01300013	Study to Evaluate the Safety and Efficacy of IV Infusion Treatment With Omecamtiv Mecarbil in Subjects With Left Ventricular Systolic Dysfunction Hospitalized for Acute Heart Failure (ATOMIC-AHF)	Phase II, Double-blind, Randomized, Placebo-controlled, Multicenter	Completed	2011-2013	Not published
NCT01380223	A Pharmacokinetic and Pharmacodynamic Study of Omecamtiv Mecarbil in Healthy Volunteers	Phase I, Double-Blind, Randomized, Four-Way Crossover, Placebo-Controlled, Safety study	Completed	2005-2006	Teerlink et al., 201128
NCT00682565	Pharmacokinetics (PK) and Tolerability of Intravenous (IV) and Oral CK-1827452 in Patients With Ischemic Cardiomyopathy and Angina	Phase II, Double-Blind, Randomized, Safety study	Completed	2008	Not published
NCT00941681	Pharmacokinetics of Oral CK-1827452 in Patients with Stable Heart Failure	Phase II, Open label, Randomized , Parallel Assignment, Pharmacokinetics study	Completed	2009	Not published
NCT00624442	A Study of CK-1827452 Infusion in Stable Heart Failure	Phase II, Double-blind, Randomized, Crossover Assignment, Safety/Efficacy Study	Completed	2007-2009	Cleland et al, 2011^35^
NCT00748579	Phase II Study of the Effect of CK-1827452 Injection on Myocardial Efficiency	Phase II, Open Label, Single Group Assignment, Pharmacokinetics/ Dynamics Study	Terminated	2008- 2009	Not published
NCT01786512	COSMIC-HF - Chronic Oral Study of Myosin Activation to Increase Contractility in Heart Failure	Phase II, Double-blind, Randomized, Placebo-controlled, Multicenter, Dose Escalation	Recruiting	2013-2015	Not published
NCT01737866	Pharmacokinetics Study of AMG 423 in Healthy Subjects and Subjects with Various Degrees of Renal Insufficiency	Phase I, Open Label, Single Group Assignment, Safety Study	Completed	2012-2013	Not published

The first one was realized by Teerlink et al. during 2005-2006^28^. This phase I, single center, double-blind, randomized, dose-escalating, placebo-controlled trial aimed to evaluate the maximum tolerated dose of OM and to investigate its pharmacodynamic and pharmacokinetic properties. 34 healthy men divided in 4 cohorts (8-10 individuals each) received 6 hours continuous intravenous infusions of increasing OM doses (0.005 mg/kg/h to 1 mg/kg/h) at one week apart. The maximum tolerated dose was established at 0.5 mg/kg/h which was well supported by 16 subjects during 6 hours, the maximum concentration being 905 ng/ml. Other findings concerning the pharmacologic properties were: mean elimination half time of 17.1-23h, clearance of 132-207 mL/h/kg and mean volume of distribution of 3.7-5.2 L/kg. Serial echographic measurements showed that EF, SV, SET time and fractional shortening increased in a dose-dependent manner.

The diastolic function seemed to be less influenced, with a decrease of ratio between early maximal ventricular filling velocity and late filling velocity (E/A), due to an improvement and prolongation of atrial contraction. The limiting effect was myocardial ischemia (moderate troponin elevation but no infarction) seen at doses higher than 0.75 mg/kg/h. This was most probably generated by a shortening in diastolic coronary filling time due to systolic prolongation. In the tolerated doses range the authors noted some more frequent pain at catheter insertion site, but without other adverse reactions. No effect on HR was observed.

Cleland et al conducted the first study in stable HFLEF patients (EF<40%, steady therapy status) which enrolled 45 patients in a double-blind, randomized, crossover, dose ranging, phase II design^35^. Only patients with sinus rhythm, glomerular filtration rate (Modification of Diet in Renal Disease -MDRD) over 35 mL/min, no cardiovascular-related hospital admission during the last 6 weeks and no Canadian Class III or IV angina were included. Divided in 5 cohorts of 8-10 individuals each, the patients received 2h, 24h or 72h escalating infusions doses (loading + maintenance) of OM in order to achieve the previously established tolerated dose range in healthy volunteers. The data analysis confirmed the directly proportional relation between plasma concentration (pC) and systolic ejection time, fractional shortening (at pC >100ng/mL) and stroke volume (at pC>200 ng/mL). The ejection fraction increased at pC>300 ng/mL and both left ventricular systolic and diastolic volumes decreased at pC>500 ng/mL. The results persisted after 24h and 72h of infusion. Atrial contraction was also prolonged, with a decrease of E/A. Interestingly, it was noted a reduction in HR which was not the case in healthy patients, and a slight decrease in QTc. Myocardial ischemia was noticed in 3 patients - one accidentally overdosed, and two others with low drug clearance, obesity and hypertension. For the remaining patients no elevation in NT-proBNP, nor troponin was observed.

The data from ATOMIC-AHF (NCT01300013) study were presented at European Society of Cardiology Congress in 2013^36^. This double-blind, randomized, placebo-controlled, multicenter, phase II study included 600 patients with decompensated systolic HF (EF<40%) - dyspnea at rest or with minimal effort resistant to diuretics and elevated BNP or NT pro-BNP. Patients were treated in 3 cohorts with 48h OM infusion targeting pC of 115, 230, 310 ng/mL respectively. The primary endpoint of dyspnea amelioration (7 points Likert Scale) was not achieved but a favorable trend was observed with higher doses and also a reduction in episodes of worsening of HF. These might be attributed to the lower pC achieved in this study comparative with the other studies. However, the previous findings in healthy volunteers and stable HF were replicated regarding the increase in SET, decrease in HR and safety issues (despite a slightly increase in troponin non related to pC).

Other phase I or II studies investigating the pharmacological properties of oral or IV OM in patients with ischemic cardiomyopathy, stable heart failure or with various degrees of renal failure were performed without published results yet (NCT01737866, NCT00682565, NCT00941681, NCT00748579). COSMIC-HF (NCT01786512) is an interesting study that may answer some questions about the future of OM but is still recruiting at this moment. Designed as a phase II, double-blind, randomized, placebo-controlled, multicenter, dose escalation study, it aims to evaluate an oral modified release (MR) OM formulation (BID) in HFLEF over 20 weeks of treatment.

Lots of hopes have been generated by OM, supported by the rather positive findings of current studies^37^. A success for OM in clinical trials might lead to a change in HF management paradigm. Despite that, there are still more questions than answers because the published studies were restricted only to a specific HF population until now. For example, it would be important to clarify the behavior of OM in patients with atrial arrhythmias or atrio-ventricular conduction abnormalities, because it seems that OM might modify atrial contractility and shorten QTc. The patients with valvular pathologies or obstructive hypertrophic cardiomyopathy should also be investigated, prolongation of SET being the marker of OM. COSMIC-HF might bring insights about OM chronic therapy and also about pharmacologic properties of oral administration.

At this moment, omecamtiv mecarbil appears to be a distinctive and unique molecule whose mechanism of action seems promising in the future therapeutic interventions. It is certain that further studies are needed to understand the pharmacodynamics and the therapeutic outcomes of this promising drug.

- **Current inotropes have serious adverse effects trough calcium- or cAMP-mediated mechanisms**
- **There aren’t any inotropic drugs FDA-approved in the last two decades**
- **Direct myosin activator OMECAMTIV MECARBIL emerges as an innovative therapy in heart failure by prolongation of systolic ejection time but further studies are necessary to validate its importance**
